# Late Lung Metastasis of a Primary Eccrine Sweat Gland Carcinoma 10 Years after Initial Surgical Treatment: The First Clinical Documentation

**DOI:** 10.1155/2013/167585

**Published:** 2013-04-28

**Authors:** R. F. Falkenstern-Ge, S. Bode-Erdmann, G. Ott, M. Wohlleber, M. Kohlhäufl

**Affiliations:** ^1^Division of Pulmonology, Klinik Schillerhoehe, Center for Pulmonology and Thoracic Surgery, Teaching Hospital of the University of Tuebingen, Solitude Street 18, 70839 Gerlingen, Germany; ^2^Division of Pathology, Robert Bosch Krankenhaus, Teaching Hospital of the University of Tuebingen, Auerbachstrasse 110, 70376 Stuttgart, Germany

## Abstract

*Background*. Sweat gland carcinoma is a rare malignancy with a high metastatic potential seen more commonly in elderly patients. The scalp is the most common site of occurrence and it usually spreads to regional lymph nodes. Liver, lungs, and bones are the most common sites of distant metastasis. Late lung metastasis of sweat gland adenocarcinoma after a time span of 5 years is extremely rare. *Aim*. We report a patient with late lung metastasis of a primary sweat gland carcinoma 10 years after initial surgical resection. *Conclusion*. Sweat gland carcinomas are rare cancers with a poor prognosis. Surgery in the form of wide local excision and lymph node dissection is the mainstay of treatment. Late pulmonary metastases with a latency of 10 years have never been reported in the literature. This is the first clinical documentation of late lung metastasis from sweat gland carcinoma with a latency period of 10 years.

## 1. Introduction

**Figure 1:**
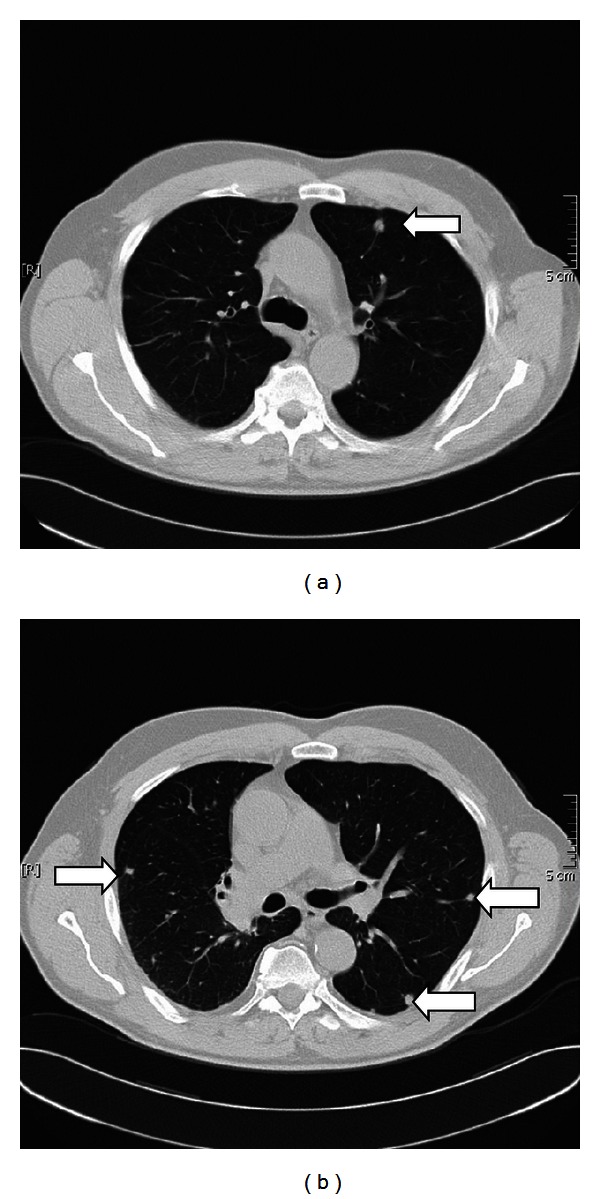
The solitary pulmonary metastasis was resected. (b) Eight months after the wedge resection, we found multiple bilateral pulmonary metastases.

**Figure 2:**
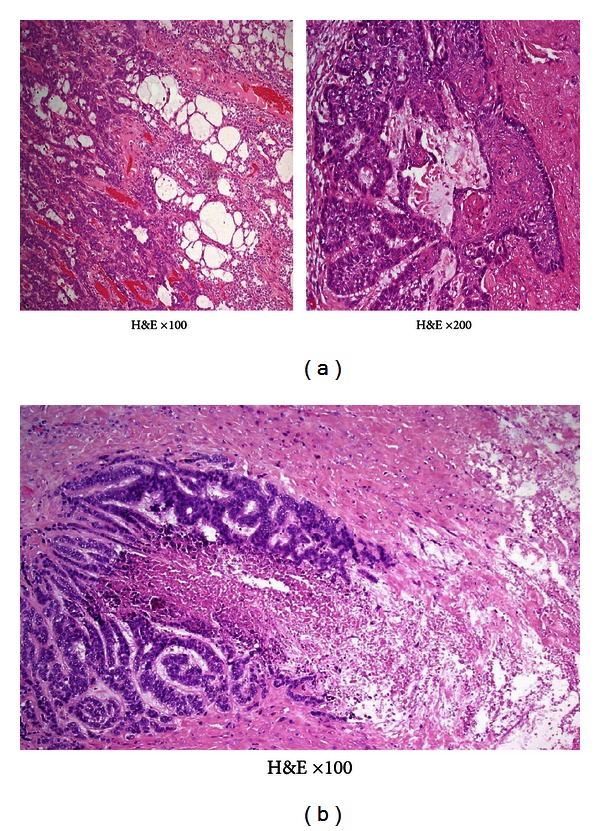


A 69-year-old man was admitted for evaluation of a solitary pulmonary nodule (1.2 cm diameter) in the left upper lobe (Figure 1(a)). The patient had a history of a sweat gland cancer in the left axilla, which was successfully resected 10 years ago. The tumor had then been classified as malignant eccrine porocarcinoma. Postsurgical followup for 10 years showed no metastasis. The new solitary pulmonary nodule was resected by surgical wedge resection, and histology was found to be compatible with a metastasis of sweat gland carcinoma (Figure 2(a)). Eight months after the initial wedge-resection, multiple bilateral pulmonary metastases were detected in a follow-up CT scan (Figure 1(b)).

## 2. Histology

The initial resection specimen from the solitary left pulmonary nodule showed infiltration by nests and islands of small ductal epithelia, sometimes with basaloid morphology (to the left), and areas of structures with glandular differentiation (to the right) (Figure 2(a), H&E ×100), eventually forming squamous nests (Figure 2(a) inset, H&E ×200). 

A later biopsy revealed in part necrotic metastases of basaloid cells organized in ribbons and strands (Figure 2(b), H&E ×100). 

Immunohistochemistry revealed positive reactions for EMA and CEA and negativity for TTF1, and the tumor was considered compatible with metastasis of a primary sweat gland adenocarcinoma. 

**Figure 3:**
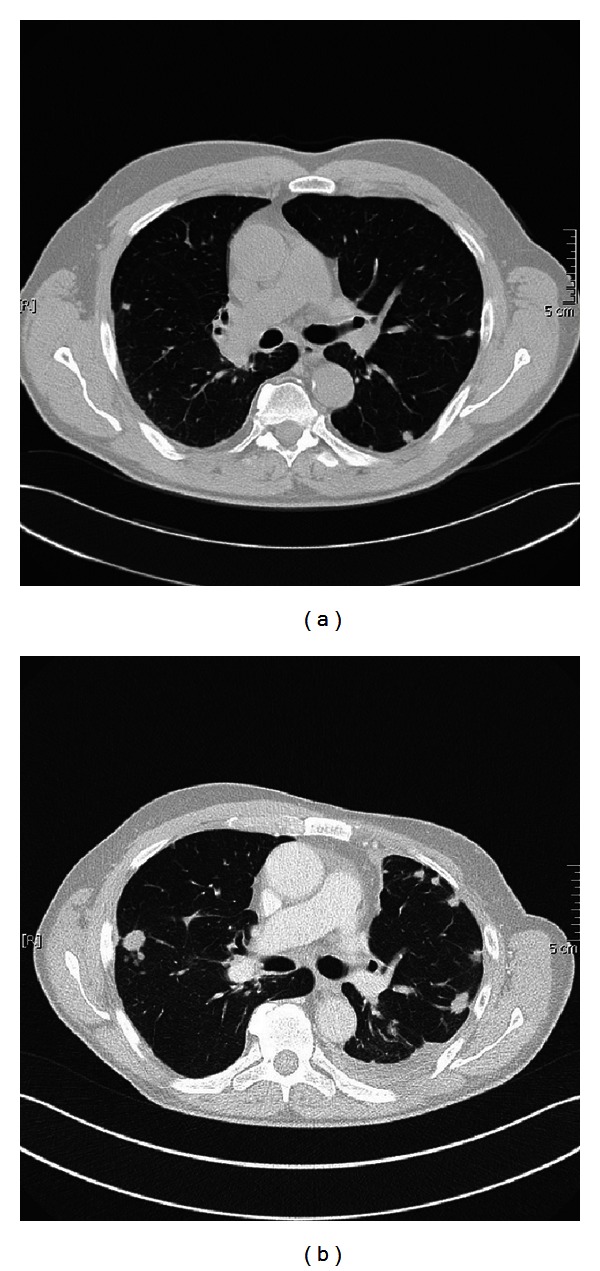
CT scan before the palliative chemotherapy with docetaxel and gemcitabine (a); the reevaluation CT scan (b) showed clear bilateral pulmonary progression after multiple cycles of palliative chemotherapies.

Because of the widespread pulmonary metastasis, metastasectomy could not be performed. Systemic chemotherapy with docetaxel was initiated; after 6 cycles of monotherapy with docetaxel, restaging showed stable disease. However, 5 months later, we observed widespread metastasis with osseous infiltration, which required palliative radiation, and second line therapy with gemcitabine was applied. The most recent restaging after 6 cycles gemcitabine showed progressive bilateral pulmonary metastasis (Figures 3(a) and 3(b)).

Due to the low performance status of the patient with severe tumor progression, the palliative chemotherapy was stopped and the patient received best supportive care.

## 3. Discussion

This is the first clinical documentation of an extremely late pulmonary recurrence of sweat gland carcinoma 10 years after successful initial resection. 

Sweat gland carcinomas are very rare malignant tumors that were first described by Cornil in 1865 [1, 2]. They have been reported to occur at various sites, including eyelids, scalp, foot digits, breast, axilla, and nose. The molecular pathogenesis is poorly understood. A low incidence of loss of heterozygosity at chromosome 17p has been noticed along with p53 alterations. These tumors are more aggressive than squamous or basal cell carcinoma [3, 4]. 

The two basic types of sweat glands in the humans are eccrine and apocrine. The eccrine glands are present everywhere, except the lips, glans penis, inner surface of the prepuce, clitoris, and labia minora. Eccrine glands are most dense on palms and soles and respond primarily to cholinergic stimuli, hence playing an important role in regulating the body temperature. The apocrine sweat glands are limited to ear canal, the eyelids, the axilla, the anogenital region and the mammary areola and are under the control of sexual hormones. However, division of sweat gland carcinomas into eccrine and apocrine groups is not clinically useful as the existing literature has not adequately subdivided and studied the separate entities well enough to make this distinction relevant for clinical purposes. An orderly progression of spread to regional lymph nodes and distant sites with metastatic adenocarcinomas can be observed and shows a poor prognosis [5]. 

The recommended treatment of all subtypes of sweat gland carcinomas is wide surgical excision along with regional lymph node dissection in the presence of clinically positive nodes. Some authors advocate prophylactic regional lymph node dissection especially in patients with recurrent lesions after wide excision or with highly undifferentiated tumors. Sweat gland carcinomas are regarded as resistant to radiotherapy. Chemotherapy has been very infrequently employed [3, 5–7]. Metastatic eccrine porocarcinoma has proven to be very resistant to many chemotherapeutic agents. The use of docetaxel in the management of this severe disease with therapeutical success was previously documented [8]. 

Prognostic factors for sweat gland carcinoma are difficult to identify, again owing to the very small number of reported cases. Prognostic factors include size, histological type, lymph node involvement, and distant metastasis.
